# Comorbidity indexing for prediction of the clinical outcome after stereotactic body radiation therapy in non-small cell lung cancer

**DOI:** 10.1186/s13014-018-1156-1

**Published:** 2018-11-03

**Authors:** Julia Dreyer, Michael Bremer, Christoph Henkenberens

**Affiliations:** Department of Radiotherapy and Special Oncology, Medical School Hannover, Carl-Neuberg-Str. 1, 30625 Hannover, Germany

## Abstract

**Purpose:**

To determine the prognostic impact of comorbidity and age in medically inoperable early-stage non-small cell lung cancer (NSCLC) treated with stereotactic body radiotherapy (SBRT) using the age-adjusted Charlson Comorbidity Index (aCCI).

**Patients and methods:**

Between November 2008 and January 2015, 196 consecutive patients with medically inoperable NSCLC were treated with SBRT at a single institution. The prescribed isocenter dose was either 60.0 Gray (Gy) in six fractions for central lung cancer or 56.25 Gy in three fractions for peripheral lung cancer. Baseline comorbidities were retrospectively retrieved according to available outclinic medical records as well as the hospital information system. The aCCI was scored for each patient and subjected according to outcome and toxicity as well as all of the single items of the aCCI and other clinical parameters using univariate and multivariate analysis.

**Results:**

Thirty-one point 6 % (62/196) of patients were deceased, of whom 17.3% (34/196) died due to lung cancer and 14.3% (28/196) due to comorbidities. The median overall survival (OS) was 15.0 months (95% CI [11.9–18.1]), whereas the median cancer-specific survival (CSS) was not reached. An aCCI ≥7 compared with an aCCI ≤6 was significantly associated with an increased risk of death (HR 1.79, 95% CI [1.02–2.80], *p* = 0.04) and cancer-specific death (HR 9.26, 95% CI [4.83–24.39], *p* < 0.001), respectively. Neither OS nor CCS were significantly associated with age, sex, side (left vs. right), lobe, localization (central vs. peripheral), packyears, TNM, or any item of the aCCI. Considering the 14.3% (28/196) of deceased patients who died due to comorbidities, aCCI ≥9 was significantly associated with non-cancer-related death (HR 3.12, 95% CI [1.22–8.33], *p* = 0.02). The observed cumulative rate of radiation pneumonitis (RP) ≥2 was 12.7% (25/196). The aCCI had no statistical association with RP.

**Conclusion:**

Advanced age and numerous comorbidities characterizing this patient population were successfully assessed using the aCCI in terms of survival. Therefore, we recommend that age and comorbidity be indexed using the aCCI as a simple scoring system for all patients treated with SBRT for lung cancer.

## Introduction

Lobectomy remains the standard of care for early-stage non-small cell lung cancer (NSCLC) in medically fit patients [1], but approximately 20% of patients are medically inoperable due to comorbidities, old age, or both [2]. Among the strategies to improve control rates, stereotactic body radiotherapy (SBRT) is the most favored. Numerous reports have indicated extremely good local control after SBRT with an excellent toxicity profile [3–5].

However, the reported overall survival rates after SBRT for early-stage NSCLC tend to be worse than local control. This has frequently been attributed to competing comorbidities because patients are treated with SBRT instead of surgery due to their comorbidities [6–9]. The choice against surgery and in favor SBRT has been found to depend on local practice [6] and patient-specific factors [7–9]. Baseline comorbidities and their prognostic impacts on the clinical outcome have not been assessed using a simple and objective comorbidity score. With this study, we aimed to make another step towards this goal. The objective of this retrospective study cohort was therefore to use the age-adjusted Charlson Comorbidity Index (aCCI) [10], as it is tempting to use given its simplicity, to investigate the impact of comorbidities on the outcome of NSCLC treated with SBRT.

## Patients and methods

### Patients

Between November 2008 and January 2015, 196 patients with medically inoperable NSCLC were treated with SBRT at a single institution. Patient were collected by reviewing the available outclinic medical records and the medical records of the hospital information system. Comorbidities were encoded using the aCCI (Table 1). The selection criteria were medically unfit for surgery or declination of surgery and staging of tumor and distant metastasis based on positron emission tomography (PET) computed tomography (CT) and biopsy of the tumor if the medical condition allowed bronchoscopy or CT guided biopsy.

**Table 1:** Age-adjusted Charlson Comorbidity Index (aCCI) [10]

Score	Comorbid condition
1	Myocardial infarction
	Congestive heart failure
	Cerebral vascular disease
	Peripheral vascular disease
	Dementia
	COPD
	Connective tissue disease
	Peptic ulcer disease
	Mild liver disease
	Age^a^
2	Diabetes
	Hemiplegia
	Moderate(Severe renal disease
	Diabetes with end-organ damage
	Solid tumor
	Leukemia
	Lymphoma
3	Moderate/severe liver disease
6	Metastatic solid tumor
	Acquired immunodeficiency syndomre

^a^1pint is added to aged 41–50 years, 2 points for those aged 51–60 years, 3 points for those 61–70 years, and 4 points for those 71 years or older

### Radiotherapy

Patients were fixed in a stereotactic body frame system with a customized vacuum pillow (Elekta, Stockholm, Sweden) using abdominal compression and free breathing. The gross tumor volume was defined based on CT findings in lung and soft tissue windows including all small spiculae. Slow scan cone beam computed tomography was performed to determine the internal target volume (ITV) until October 2014, and 4-dimensional CT was used after that. We added a margin of 4 mm in all directions to the ITV to define the planning target volume (PTV). SBRT treatment planning was conducted with Oncentra Masterplan (Elekta, Stockholm, Sweden). Irradiation was performed as multifield irradiation using a linac accelerator every second day. The prescribed isocenter dose for peripheral located tumors was 18.75 Gy (PTV border covered by the 67% isodose), and the total dose was 56.25 in three fractions. Centrally located tumors usually received an isocenter dose of 7.5 Gy (PTV boarder covered by the 80% isodose), and the total dose was 60.0 Gy. Dosimetric calculation was conducted using a pencil beam algorithm with heterogeneity correction. The constraints for RT planning are described elsewhere [5, 11]. In some patients, the dose was individually adjusted to the dose exposure of organs at risk. The detailed patient characteristics are summarized in Table 2.

**Table 2:** Patient characteristics

	*n* = 196 (%); median	range
Sex		
female	73 (37.1)	
male	123 (62.9)	
Medically inoperable	182 (92.8)	
Medically operable	14 (7.2)	
Localization		
-central	83 (42.3)	
-peripheral	113 (57.7)	
Side		
-left	86 (43.9)	
-right	110 (56.1)	
Grading (G)		
G1	7 (3.6)	
G2	74 (37.8)	
G3	42 (21.4)	
Stage according to UICC (7th edition)		
-I	113 (57.7)	
-II	68 (34.6)	
-IIIa	15 (7.7)	
Histology		
-Adenocarcinoma	49 (39.9)	
-Squamous cell carcinoma	71 (57.7)	
-Large cell carcinoma	3 (2.4)	
-No biopsy due to comorbidities	73 (37.2)	
Age	67	29–86
0–50	6 (3.1)	
50–65	63 (32.1)	
66–80	101 (51.5)	
> 80	26 (13.3)	
aCCI	7	3–16
0–3	4 (2.0)	
4–6	63 (32.1)	
7–9	62 (31.6)	
10–12	44 (22.4)	
> 12	23 (11.9)	
Hypertension	119 (60.7)	
Diabetes with or without end-organ damage	52 (26.6)	
Moderate/severe renal damage	63 (32.1)	
COPD	167 (85.2)	
-Gold 1 + 2	51 (26.0)	
-Gold 3	61 (31.1)	
-Gold 4	55 (28.1)	
Peripheral vascular disease	49 (25)	
Myocardial infarction	31 (15.8)	
Congestive heart failure	71 (36.2)	
Cerebral vascular disease	13 (6.6)	
Mild liver disease	9 (4.5)	
Isocenter Dose		
-peripheral tumor	18.75	18–20
-central tumor	7.5	7–9
Packyears	40	0–120

### Follow-up

Follow-up visits were performed every 3 months and included CT of the chest and abdomen. ^18^F-fluorodeoxyglucose positron emission tomography (FDG-PET) was performed when CT was suspicious for relapse. The date of relapse was determined as the date when FDG-PET was assessed as positive for local and/or distant relapse by experienced nuclear physicians or when biopsy proved relapse in medically fit patients. Overall survival (OS) was defined as the period from the last day of SBRT to the date of death from any cause. Lung cancer death was defined as death resulting from the progression of lung cancer (local and/or distant), and non-lung cancer death was defined as death of any other cause due to comorbidities. Locoregional relapse was defined as any relapse within the lung or mediastinum, and distant metastases were defined as lung cancer lesions outside the lung and mediastinum.

### Toxicity

Toxicity was assessed weekly during SBRT by anamnesis and physical examination. Acute toxicity was defined from the start of SBRT up to 90 days after the last day of irradiation and was graded according to the Common Toxicity for Criteria Adverse Events (CTCAE V 4.0) [12]. Late toxicity was defined as symptoms > 90 days after the last fraction of SBRT and was classified according to the Late Effects on Normal Tissue-Subjective, Objective, Management scales (LENT-SOMA) [13].

### Statistics

The outcomes were statistically assessed using Kaplan Meier analysis with log-rank test and Cox regression analysis.

Toxicity was statistically assessed with univariate analyses using the Chi-squared-test for non-parametric parameters and Student’s *t*-test for parametric parameters. Multivariate logistic regression analysis included all significant parameter from the univariate analysis using backwards elimination to determine the parameters that contributed the most to toxicity. The factors evaluated were age, sex, histology, grading, side, localization, TNM stage, packyears, aCCI and all single items of the aCCI. Statistical analysis was performed with a commercially available software package (SPSS V.24, IBM, Armonk, NY, USA).

## Results

### Outcome

The median overall survival was 15.0 (3.0–64.0) months for all patients and the median follow-up was 24.0 months (6–64.0) for patients who were alive (66.8% [131/196]. Concerning all patients, 31.6% (62/196) were deceased and 1.6% (3/196) were lost to follow-up. Seventeen point 3 % (34/196) of patients died due to lung cancer, 6.1% (12/196) due to locoregional failure and 11.2% (22/196) due to distant extrapulmonary metastases. Furthermore, 14.3% (28/196) of patients died due to comorbidities. The detailed results are shown in Table 3.

**Table 3:** Descriptive outcome analysis

Status		*n*	%
Alive		131	66.8
Deceased		62	31.6
Unknown		3	1.6
Death from lung cancer		34	17.3
Locoregional failure		12	6.2
Distant progression		22	11.2
Death from comorbidities		28	14.3
cardiovascular		8	4.1
lung		6	3.1
infection		4	2.0
stroke		3	1.5
other		7	3.6

**Fig. 1:**
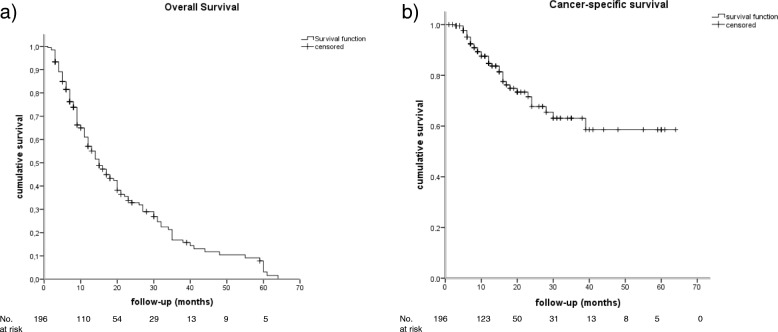
Outcome. Kaplan-Meier curves of the Overall Survival (**a**) and the Cancer-specific survival (**b**)

**Fig. 2:**
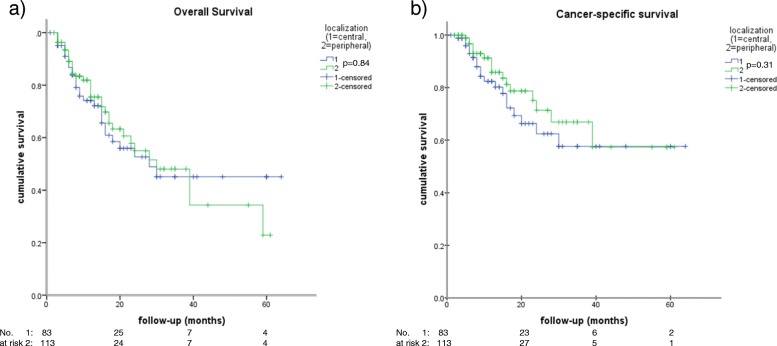
Kaplan-Meier curves of Overall (**a**) and Cancer-specific survival (**b**) comparing peripheral vs. central tumor localization showing no significant difference (*p* = 0.84 and *p* < 0.31)

**Fig. 3:**
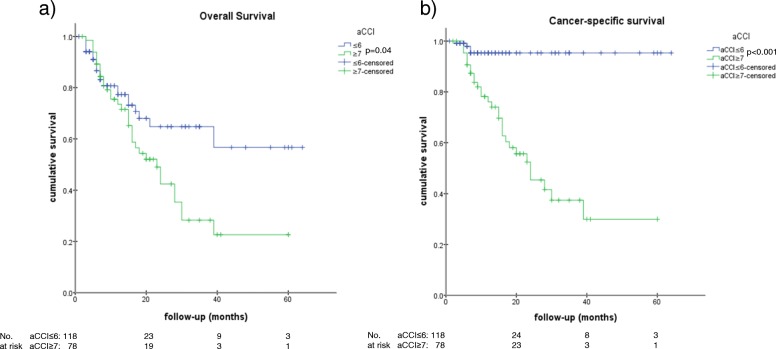
Kaplan-Meier curves Overall (**a**) and cancer-specific survival (**b**) comparing aCCI of ≤6 with aCCI ≥7 showing a significant reduced survival rates (*p* = 0.04 and *p* < 0.001)

The median OS was 15.0 months (95% CI [11.9–18.1], Fig. 1a), whereas the median cancer-specific survival (CSS) was not reached (Fig. 1b). In addition, 45.2% (28/62) of the deceased patients died from competing comorbidities and 54.8% (34/62) from lung cancer. Neither OS (Fig. 2a) nor CCS (Fig. 2b) was significantly worse for central tumors compared with peripheral tumors (HR 1.05, 95% CI [0.64–1.70], *p* = 0.85; HR 1.40, 95% CI [0.73–2.70], *p* = 0.31). Considering the survival of the presented patient cohort divided by the median aCCI of 7, aCCI ≥7 compared with a aCCI of ≤6 was found to be significantly associated with an increased hazard for death (HR 1.79, 95%CI [1.02–2.80],*p* = 0.04) and cancer-specific death (HR 9.26, 95% CI [4.83–24.39], *p* < 0.001), respectively. The corresponding Kaplan Meier curves of the OS and CCS are shown in Fig. 3. Neither OS nor CCS was significantly associated with age, sex, side (left vs. right), lobe, localization (central vs. peripheral), packyears, TNM, or any item of the aCCI. Considering the 14.3% (28/196) of deceased patients who died due to comorbidities, aCCI ≥9 was significantly associated with non-cancer-related death (HR 3.12, 95% CI [1.22–8.33], *p* = 0.02).

### Toxicity

Due to the low number of events, the frequencies of acute and late toxicity were assessed cumulatively. We observed no fatal toxicity related to SBRT.

Radiation pneumonitis (RP) of grade 1 occurred in 34.7% (68/196), of grade 2 in 11.2% (22/196), of grade 3 in 1.0% (2/196), of grade 4 in 0.5% (1/196) and of grade 5 in 0% (0/196) of patients, respectively. This resulted in a cumulative RP ≥2 rate of 12.7% (25/196). Univariate analysis revealed that tumors located on the right lung side (*p* = 0.01) were associated with clinically relevant RP ≥ grade 2. Age, sex, lobe, localization (central vs. peripheral), packyears, TNM, aCCI nor any item of the aCCI were statistically associated with RP ≥ grade 2.

In total, 7.7% (15/196) of patients developed a radiation esophagitis (RE) grade ≥ 2. No patients (0/113) with peripheral tumors developed an RE grade ≥ 2, whereas 16.9% (14/83) of the patients with central tumors developed acute RE grade 2, and 1.2% (1/83) acute RE grade 3, respectively. No late RE ≥ grade 1 was observed. Univariate statistical analysis revealed no significant parameters associated with RE. Furthermore, in 2.1% (4/196), a mild chest wall toxicity (CWT) grade 1 with no need for narcotics was observed. No CWT ≥ grade 2 was observed. In addition, none of the assessed toxicities (RP, RE, CWT) were associated with items of the aCCI.

## Discussion

To our best knowledge, the presented study represents the largest early-stage lung cancer population treated with SBRT to quantify the impact of baseline co-morbidities on the clinical outcome.

The observed median overall survival of 15.0 months was low, although this rate is consistent with other studies [14–16]. Convincing data suggest that poor survival–despite high local control rates–is attributed to advanced age and competing comorbidities because subgroup analysis revealed that medically operable patients treated with SBRT had a much higher survival than medically inoperable patients treated with SBRT [17–20], which was recently confirmed in a prospective single-arm phase 2 study conducted by the NRG Oncology Radiation Therapy Oncology Group [21]. Although a large randomized trial comparing surgery with SBRT for medically operable early stage NSCLC does not exist; SBRT is a good alternative to surgery [17–21] with lower direct medical costs and better quality-adjusted life expectancies [22]. Furthermore, Eguchi et al. showed in a competing risks analysis of curative-intent resection of stage I lung cancer that high age was a significant parameter for worse short-term outcome and 1-point increase of the CCI (not age-adjusted) decreased the overall survival by 14% [23].

Therefore, it is lucid to assume that reported data from lung cancer patients treated with SBRT are biased by not precisely recorded high age, number and severity of comorbidities and not by the technique of SBRT itself, which may compromise survival. Therefore, the age-adjusted Charlson Comorbidity Index (aCCI) was used in this study to assess the prognostic significance of age and co-morbidity. The improved survival of early-stage NSCLC –particularly in medically unfit patients- is related to the widespread adoption of SBRT to the clinical routine [24–27]. A direct comparison of the survival between lobectomy and SBRT is limited by the inherent unmeasured biases of databases warranting dedicated prospective trials [24, 25].

The results of our analysis provide some reassurance that it is indeed advanced age coupled with competing baseline comorbidity rather than overlooked treatment-related mortality that is largely responsible for the low observed OS and CSS rates post-SBRT. Patients with aCCI ≥7 had a significantly increased hazard for death and cancer-specific death compared with patients with an aCCI ≤6. The median aCCI was 7, suggesting usually three other competing comorbidities for a patient cohort with a median age of 70. This is considerably higher than in other reports [14, 28, 29], although some studies do not use the age-adjusted CCI [14, 29]. Nevertheless, in our analysis neither age nor any of the single comorbidities of the aCCI were significantly associated with outcome suggesting that an age-adjusted comorbidity index, such as the aCCI, should be used instead of an index that does not adjust for age because age is generally associated with poor survival after SBRT for lung cancer [30]. The CCI in general does not graduate severity of comorbidities in a precise way, and Extermann et al. were cautious about the CCI due to its tendency to underrate the functional status of older cancer patients [31]. In the particular case of SBRT, lung cancer patients are often not eligible for surgery due to chronic obstructive pulmonary disease (COPD) Gold III or Gold IV, which is the major reason for allocating to SBRT. The aCCI distinguishes chronic pulmonary disease as yes or no but does not take into account that patients with severe COPD Gold III or IV have a substantially reduced life expectancy, even without lung cancer [32, 33]. The severity of COPD classified according to the Gold criteria was not assessed, although that might have been a confounder that influenced survival analysis using aCCI. An alternative score is the Cumulative Illness Rating Scale for Geriatrics (CIRS-G), which allows a subjective grading of severity of comorbidities in elderly patients [34, 35], but the CIRS-G has never been used in a large patient cohort with mainly medically inoperable lung cancer patients treated with SBRT [20]. The CIRS-G is much more complex, more labor-intensive and less user-friendly than the aCCI because the user of the CIRS-G needs complex multidisciplinary knowledge, and sometimes the CIRS-G even requires further medical consultation [20, 34, 35]. Therefore, the aCCI is a more applicable and faster scoring system than the CIRS-G.

RP is considered to be the most important toxicity with rates of RP ≥2 ranging from 9 to 28% [36]. We observed no abnormally increased rate of clinically relevant RP ≥2 of 12.7% in the presented patient collective with mainly multimorbid patients. Several risk factors, such as age, sex, severity of COPD, baseline lung function and smoking status, have been reported with controversial results [22, 37–39]. Statistical analyses showed that tumor location on the right lung was associated with RP ≥2, which was also observed by Chaudari et al. [37]. Basically, it can be assumed that the small patient cohorts and the low incidence of RP have introduced bias into the statistical results, and thus, the results of statistical analyses have to be interpreted with caution. Therefore, we cannot rule out that the statistical association with RP ≥2 and tumor on the right lung side might be a random result.

Some limitations of this study should be acknowledged. First, its retrospective character has inherent limitations and might have introduced a selection bias. Second, this study included a selected cohort with mainly medically inoperable patients. Therefore, caution should be applied when transferring the observed results to medically fit and operable patients. Third, the aCCI certainly does not include all outcome relevant comorbidities and does not grade comorbidities according to their severity in a precise way. Additionally, the aCCI was not developed specifically for carcinoma patients, but is a more general tool to estimate the prognosis of patients. Fourth, this study is based on clinical parameters, although dosimetric parameters also have impact on outcome and side effects [5, 22, 40, 41]. Fifth, the follow-up period of 24 months for patients who were alive and the number of deceased patients (cancer related and non-cancer related death) might be insufficient to make definitive statements about long-term cancer survival and survival of comorbidities. Therefore we cannot predict long-term outcome and claim that we successfully assessed age and numerous comorbidities in general. Ideally, there would be a validation cohort for this purpose.

Nevertheless, the observed results are robust and the aCCI was a simple tool for estimation of prognosis in medically unfit patients.

## Conclusion

The results of the present study indicate that SBRT for early stage lung cancer is a well-tolerated treatment modality that offers long-term tumor control. The advanced age and numerous comorbidities characterizing this patient population were successfully assessed with the aCCI in terms of survival. Therefore, we recommend that age and comorbidity should be indexed using the aCCI as a simple score for all patients treated with SBRT for lung cancer.

## Abbreviations

aCCI: Age-adjusted Charlson Comorbidity Index
CIRS-G: Cumulative Illness Rating Scale for Geriatrics
COPD: Chronic obstructive pulmonary disease
CSS: Cancer-specific survival
CT: Computed tomography
CTCAE: Common Toxicity for Criteria Adverse Events
CWT: Chest wall toxicity
FDG: ^18^F-fluorodeoxyglucose
G: Grading
Gy: Gray
ITV: Internal target volume
LENT-SOMA: Late Effects on Normal Tissue-subjective, objective, management scales
NSCLC: Non-small cell lung cancer
OS: Overall survival
PET: Positron emission tomography
PTV: Planning target volume
RE: Radiation esophagitis
RP: Radiation pneumonitis
SBRT: Stereotactic body radiotherapy
